# Multipathway synergy promotes testicular transition from growth to spermatogenesis in early-puberty goats

**DOI:** 10.1186/s12864-020-6767-x

**Published:** 2020-05-25

**Authors:** Dongdong Bo, Xunping Jiang, Guiqiong Liu, Feng Xu, Ruixue Hu, Teketay Wassie, Yuqing Chong, Sohail Ahmed, Chenhui Liu, Shishay Girmay

**Affiliations:** 1 Laboratory of Small Ruminant Genetics, Breeding and Reproduction, College of Animal Science and Technology, Huazhong Agricultural University, Wuhan, 430070 People’s Republic of China; 2 Key Laboratory of Agricultural Animal Genetics, Breeding and Reproduction, Ministry of Education, Wuhan, 430070 People’s Republic of China

**Keywords:** Early puberty, Phase transition, Testicular growth, Spermatogenesis, Transcriptome

## Abstract

**Background:**

The microscopic process of postnatal testicular development in early-puberty animals is poorly understood. Therefore, in this study, 21 male Yiling goats with average ages of 0, 30, 60, 90, 120, 150 and 180 days old (each age group comprised three goats) were used to study the changes in organs, tissues and transcriptomes during postnatal testicle development to obtain a broad and deep insight into the dynamic process of testicular transition from growth to spermatogenesis in early-puberty animals.

**Results:**

The inflection point of testicular weight was at 119 days postpartum (dpp), and the testicular weight increased rapidly from 119 dpp to 150 dpp. Spermatozoa were observed in the testis at 90 dpp by using haematoxylin–eosin staining. We found from the transcriptome analysis of testes that the testicular development of Yiling goat from birth to 180 dpp experienced three stages, namely, growth, transition and spermatogenesis stages. The goats in the testicular growth stage (0–60 dpp) showed a high expression of growth-related genes in neurogenesis, angiogenesis and cell junction, and a low expression of spermatogenesis-related genes. The goats aged 60–120 dpp were in the transitional stage which had a gradually decreased growth-related gene transcription levels and increased spermatogenesis-related gene transcription levels. The goats aged 120–180 dpp were in the spermatogenesis stage. At this stage, highly expressed spermatogenesis-related genes, downregulated testicular growth- and immune-related genes and a shift in the focus of testicular development into spermatogenesis were observed. Additionally, we found several novel hub genes*,* which may play key roles in spermatogenesis, androgen synthesis and secretion, angiogenesis, cell junction and neurogenesis. Moreover, the results of this study were compared with previous studies on goat or other species, and some gene expression patterns shared in early-puberty mammals were discovered.

**Conclusions:**

The postnatal development of the testis undergoes a process of transition from organ growth to spermatogenesis. During this process, spermatogenesis-related genes are upregulated, whereas neurogenesis-, angiogenesis-, cell junction-, muscle- and immune-related genes are downregulated. In conclusion, the multipathway synergy promotes testicular transition from growth to spermatogenesis in early-puberty goats and may be a common rule shared by mammals.

## Background

Puberty, a complex biological process of sexual development, involves changes due to heredity, nutrition, environment and other factors. Unlike the developmental dysplasia caused by precocious puberty in humans, the early onset of puberty in livestock can reduce the age at the first litter and prolong the reproductive life to a certain extent [1]. Moreover, the testis plays an important role in androgen secretion and spermatogenesis. Therefore, studying testicular development in early-puberty animals is the key to understand the mechanism of early puberty.

The mutations of certain key genes in the hypothalamus–pituitary–gonad axis (such as *KISS1*, *GPR54*, *GnRH*, *GnRHR* and *LHR*) and some neuroendocrine centre genes (such as *MKRN3* [2], *DLK1* [3] and *LIN28B* [4]) cause pathological precocious puberty. However, the molecular genetic basis of early puberty remains controversial. Particularly, the microscopic process of postnatal testicular development in early-puberty animals is still poorly understood.

In this study, the Yiling goats, a native Chinese breed with early puberty, are used to investigate the dynamic changes in testicles at the organ, tissue and transcriptome levels. This study aims to (1) describe the postnatal testicular development in goats, (2) elucidate the genes and pathways that lead to the transition of testicles from growth to spermatogenesis and (3) clarify the dynamic expression of these genes and pathways to provide basic facts for the process of early puberty.

## Results

### Postnatal growth model of testis

**Fig. 1:**
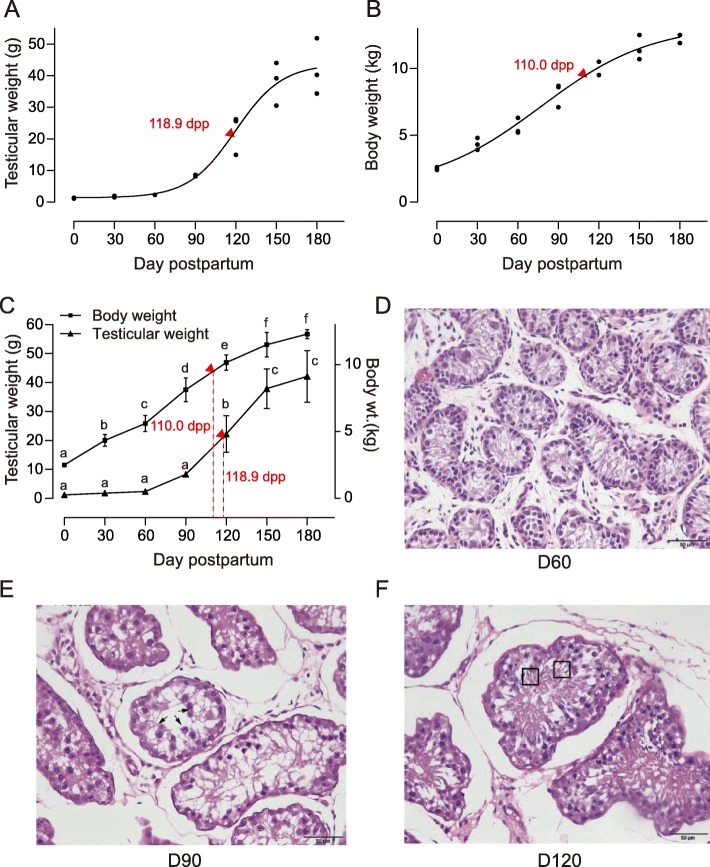
Body growth and testicular development of Yiling goat. **a** Fitting of testicular growth. The model for testicular growth was *W*_*t*_ = 44.5529/(1 + 440.4 × e^− 0.0512*d*^). *W*_*t*_ represents testicular weight, and *d* represents the number of days postpartum (dpp). The age of inflection point was 118.9 dpp. **b** Fitting of body growth. The model for body growth was *W*_*b*_ = 13.7439/(1 + 4.5074 × e^− 0.0208*d*^). *W*_*b*_ represents body weight, and *d* represents the dpp. The age of inflection point was 110 dpp. **c** Body weight and testicular weight of Yiling goat. Mean ± SE; means with different letters are significantly different among ages (*P* < 0.05). **d**, **e**, **f** Light micrographs of the testis in the Yiling goats at 60 dpp (D, D60), 90 dpp (E, D90) and 120 dpp (F, D120). At 60 dpp (D, D60), no spermatozoa were observed in the seminiferous tubules. At 90 dpp (E, D90), spermatocytes (arrowheads) were observed in the seminiferous tubules. After 90 dpp (F, D120), seminiferous tubules developed larger and many spermatozoa (square area) were seen constantly. HE staining, magnification 40×. Bar indicates 50 μm

The logistic growth model was used to fit the relationship of age with the body and the testicular weights. Results indicated that the model for testicular growth was *W*_*t*_ = 44.5529/(1 + 440.4 × e^− 0.0512*d*^). *W*_*t*_ represents testicular weight, and *d* represents the days postpartum (dpp). The age of inflection point was 118.9 dpp. No significant difference was observed in testicular weight from 0 dpp to 90 dpp (*P* > 0.05), and the testicular weight significantly increased from 120 dpp to 150 dpp (*P* < 0.05). The increment was not significant (*P* > 0.05) after 150 dpp (Fig. 1a).

The model for body growth was *W*_*b*_ = 13.7439/(1 + 4.5074 × e^− 0.0208*d*^). *W*_*b*_ represents body weight, and *d* represents the dpp. The age of inflection point was 110 dpp. A period of rapid growth occurred from 0 dpp to 110 dpp, and the increment in body weight slowed down from 110 dpp onwards. Similar to testicular growth, body growth entered the plateau stage at about 150 dpp, and no significant change (*P* > 0.05) occurred from 150 dpp to 180 dpp (Fig. 1b).

The inflection point of the testicular weight was 8.9 days later compared with that of the body weight. This finding indicated that the body weight increased before the initiation of testicular growth (Fig. 1c).

Testicular tissues were obtained, sectioned and stained with haematoxylin and eosin (HE) to observe their development. A small cross-sectional area of seminiferous tubules, thin spermatogenic epithelium and azoospermia were observed at 60 dpp (Fig. 1d, D60). At 90 dpp, a large cross-sectional area of seminiferous tubules, thick seminiferous epithelium and evident spermatozoa were observed (Fig. 1e, D90). At 120 dpp, enlarged seminiferous tubules, thickened spermatogenic epithelium and mature sperms were visible (Fig. 1f, D120).

### The expression profiles of genes in testis

**Fig. 2:**
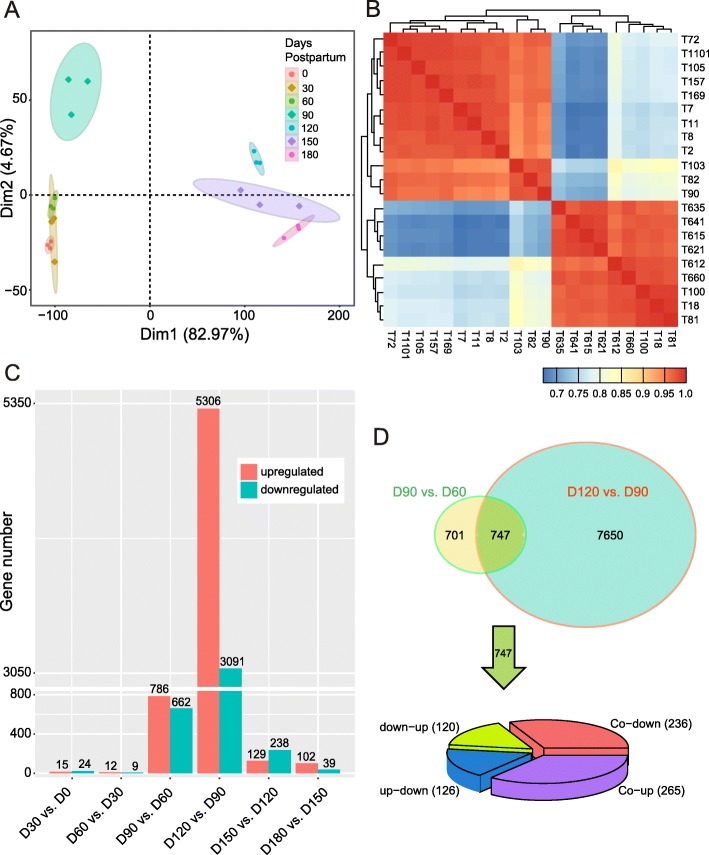
The expression profiles of genes. **a** PCA of 21-pair distinct samples across the seven ages based on normalized mRNAs expression level. The samples were grouped by age. The expression pattern of genes can be divided into three different clusters. The expression profiles of the samples at 90 dpp were separable from those at 0–60 dpp and 120–180 dpp. **b** Heat map of correlation coefficient for 21 samples based on the gene expression level. The samples were grouped by hierarchical clustering, and the dendrogram was not shown. Information of samples was shown in Additional file 8: Table S20. **c** Bar plot presentation of DEGs between neighboring age groups. **d** The Venn diagram shows the numbers of DEGs in D90 vs. D60 and D120 vs. D90. The total and overlay numbers of DEGs are 9098 and 747 respectively. The pie chart shows the changes in the common 747 DEGs

The testicular tissues of Yiling goats were sequenced for transcriptome analysis to investigate the expression profiles of genes during testicular development in early-puberty goats. The principal component analysis (PCA) demonstrated that the samples obtained at 0, 30 and 60 dpp formed a distinctive cluster, and the samples obtained at 120, 150 and 180 dpp formed another cluster. The expression profiles of the samples at 90 dpp were different from these two clusters (Fig. 2a). This result was confirmed by correlation analysis (Fig. 2b).

The differentially expressed genes (DEGs) between any two adjacent age groups (0, 30, 60, 90, 120, 150 and 180 dpp) were identified as having *q* < 0.05 and |fold change| ≥ 2 (Fig. 2c). Very few DEGs were expressed from birth to 30 dpp (15 upregulated and 24 downregulated genes), from 30 dpp to 60 dpp (12 upregulated and 9 downregulated genes) and from 150 dpp to 180 dpp (102 upregulated and 39 downregulated genes). However, the expression of genes changed the most in the 90–120 dpp stage (5306 upregulated and 3091 downregulated genes), followed by the 60–90 (786 upregulated and 662 downregulated genes) and the 120–150 (129 upregulated and 238 downregulated genes) dpp stages. Interestingly, amongst the three stages with the greatest changes in gene expression, the 90–120 dpp stage was observed with more upregulated genes than downregulated genes. Moreover, the 60–90 dpp stage was observed with slightly more upregulated genes than downregulated genes, and the 120–150 dpp stage was observed with slightly more downregulated genes than upregulated genes.

The results shown in Fig. 2d indicated that 1448 and 8397 genes were differentially expressed in the stages of 60–90 (D90 vs. D60) and 90–120 (D120 vs. D90) dpp, respectively. Moreover, 747 DEGs overlapped. Amongst these 747 genes, 265 were upregulated, and 236 were downregulated. 126 genes were upregulated at 90 dpp but downregulated at 120 dpp, and 120 genes were downregulated at 90 dpp but upregulated at 120 dpp.

### Function of DEGs during testicular development in the puberty stages

**Fig. 3:**
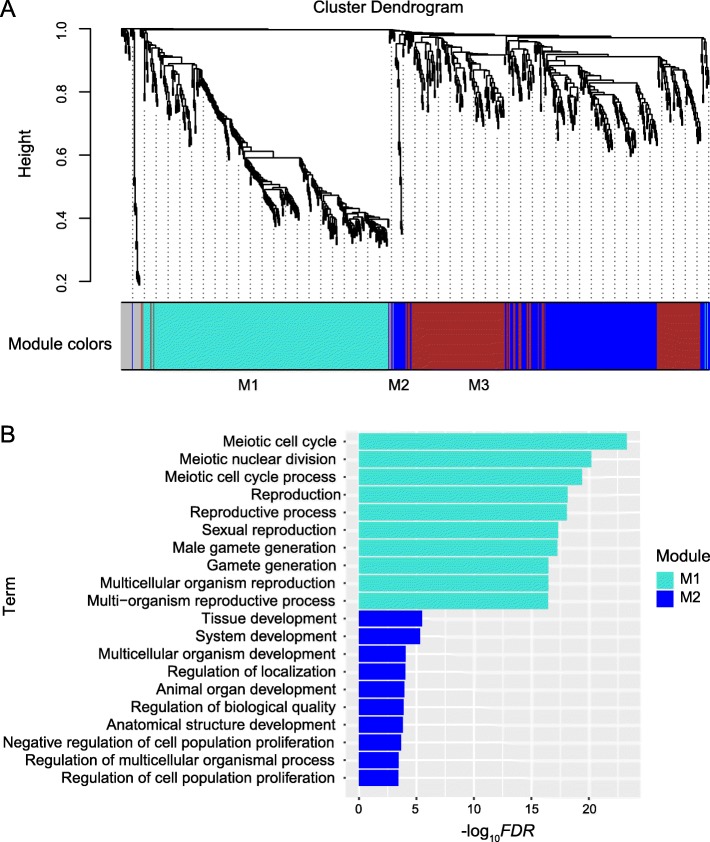
Gene modules identified by WGCNA and functional enrichment of upregulated genes of D90 vs. D60. **a** Hierarchical cluster dendrogram of upregulated genes of D90 vs. D60 obtained by clustering the dissimilarity based on consensus topological overlap. Modules corresponding to branches were labeled with colors indicated by the color bands underneath the tree. A total of three modules were identified. **b** Top ten of functional enrichment results for each module. The top ten terms with the lowest *FDR* were shown in the figure

In accordance with the growth (Fig. 1) and gene expression profile (Fig. 2) of testis in present study, the key periods of phase transition of testicular development were 60–90 and 90–120 dpp. Therefore, the weighted gene co-expression network analysis (WGCNA) method was used to cluster the expression patterns of the upregulated and downregulated genes of D90 vs. D60 and D120 vs. D90, in order to characterise the biological functions of genes in phase transition (Fig. 3a, Additional file 1: Tables S1–S9).

The upregulated genes at 90 dpp were clustered into three modules (i.e. M1, M2 and M3; Fig. 3a, Additional file 1: Tables S1–S3). For M1 (turquoise, 328 genes; Additional file 1: Table S1), the spermatogenesis-associated gene ontology (GO) terms, including *synaptonemal complex assembly*, *meiotic sister chromatid cohesion*, *chromosome localization to nuclear envelope involved in homologous chromosome segregation*, *telomere localization*, *male meiosis I*, *motile cilium assembly*, *oocyte development*, *axoneme assembly*, *spermatid development* and *fertilization*, were enriched (Additional file 2: Table S10). For M2 (blue, 214 genes; Additional file 1: Table S2), the terms *tolerance induction to self-antigen*, *positive regulation of regulatory T-cell differentiation* and *negative regulation of interleukin-17 production*, which may indicate the formation of immune-privileged environment in seminiferous tubules, were enriched (Additional file 2: Table S11). Moreover, the terms involved in the negative regulation of the development of muscles tissues, such as *negative regulation of skeletal muscle tissue development* and *negative regulation of vascular smooth muscle cell proliferation*, were enriched. Similar to those in M1, meiosis-related terms, such as *positive regulation of mitotic nuclear division*, *G1/S transition of mitotic cell cycle* and *cell cycle arrest*, were enriched in M2. The terms *positive regulation of oxidoreductase activity*, *cellular response to hypoxia* and *cellular response to decreased oxygen levels* were also enriched (Additional file 2: Table S11).

The downregulated genes at 90 dpp were clustered into two modules (M4 and M5; Additional file 1: Tables S4 and S5). For M4 (turquoise, 546 genes; Additional file 1: Table S4), the GO terms associated with cell adhesion, such as *cell–matrix adhesion*, *cell–substrate adhesion* and *cell adhesion,* were enriched. Moreover, the genes involved in morphogenesis and neurogenesis were downregulated at 90 dpp (Additional file 2: Table S12, Additional file 3: Figure S1).

The upregulated genes at 120 dpp were clustered into only one module (M6, 5116 genes; Additional file 1: Table S6). The functional enrichment analysis of the upregulated genes at 120 dpp identified diverse spermatogenesis-related genes which were grouped in accordance with the hierarchical relations and similarity amongst enriched terms. These genes were grouped as synapsis (grouped as *synaptonemal complex organization, chromosome organization, chromosome organization involved in meiotic cell cycle* and *homologous chromosome segregation*), spermatid nucleus differentiation (grouped as *cellular process involved in reproduction in multicellular organism*, *spermatid development* and *spermatid differentiation*), sperm cilium growth (grouped as *axonemal dynein complex assembly*, *motile cilium assembly*, *flagellated sperm motility*, *cilium movement* and *protein localization to cilium*) and sperm–egg binding (Additional file 2: Table S13, Additional file 4: Figure S2). In addition, we also identified the upregulation of genes involved in the metabolism of piRNA (*piRNA metabolism process*; Additional file 2: Table S13), an important post-transcriptional regulator of spermatogenesis.

The downregulated genes at 120 dpp were clustered into three modules (M7, M8 and M9; Additional file 1: Tables S7–S9). The genes in M7 (turquoise; Additional file 1: Table S7, Additional file 5: Figure S3A) were enriched in the GO subcategories related to cell junction and tissue development (Additional file 2: Table S14). In addition, a variety of GO terms important for immunity, including *positive regulation of leukocyte chemotaxis*, *adaptive immune response*, *regulation of T-cell activation*, *positive regulation of immune response* and *leukocyte activation*, was found (Additional file 2: Table S14). The GO terms associated with epithelial development were enriched for the genes in M8 (Additional file 2: Table S15; Additional file 5: Figure S3).

**Fig. 4:**
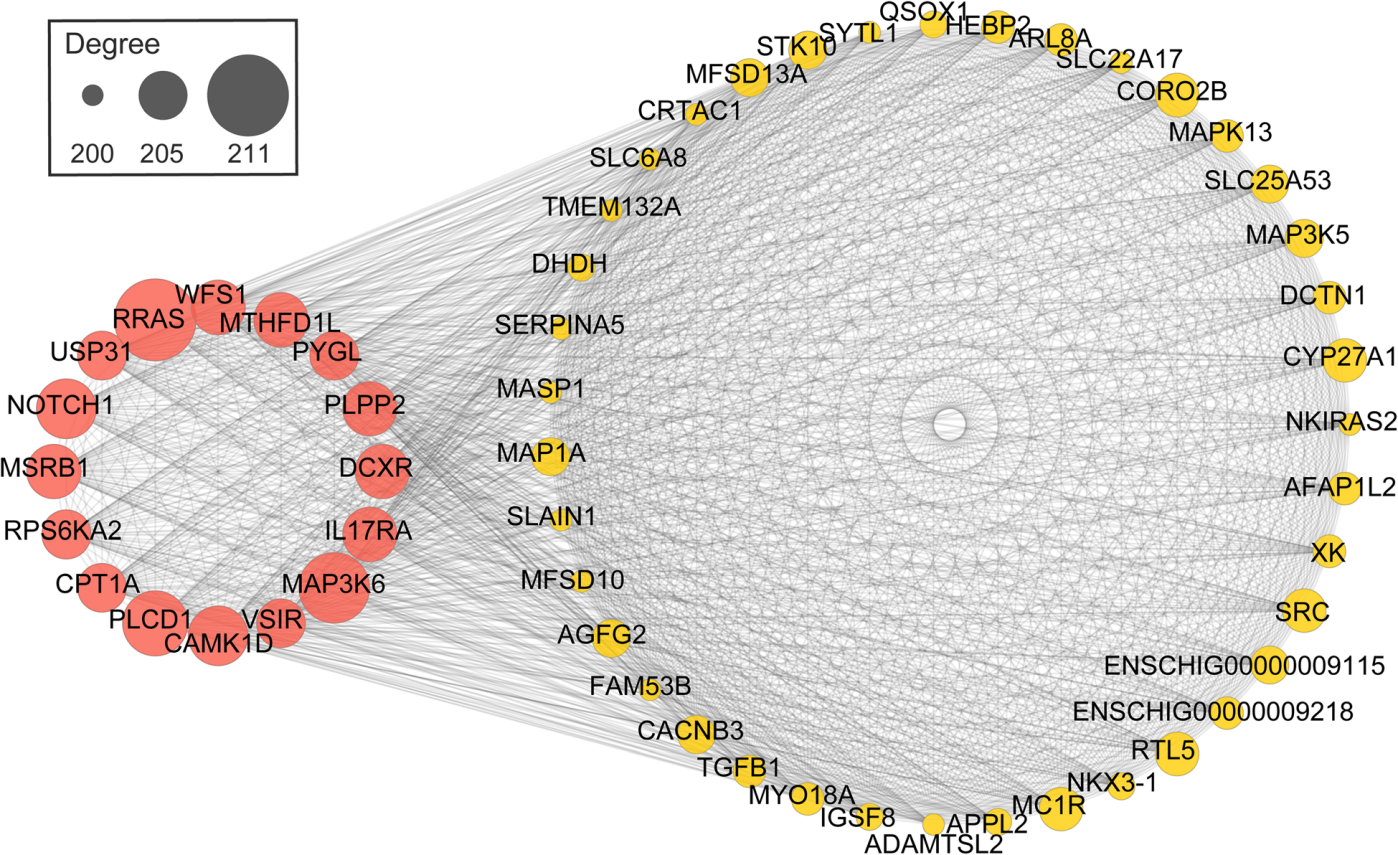
Co-expression network diagram of genes in M2. The co-expression network was generated by WGCNA analysis. A total number of 214 candidate genes was considered for the analysis and the top 55 with degree higher than 200 was shown. Proteins genes are represented as nodes. Genes with degree higher than 205 were considered as hub genes

The co-expression networks of top-ranked genes for all the modules (M1–M9) were constructed. The hub genes are shown in Fig. 4 and Table 1.

**Table 1:** The information of hub genes in each module

Module	Expression	Hub genes
M1	upregulated at 90 dpp	*MEIOB, RAD21L1, RNF181, SLC25A31, STAG3, TDRKH, ART3, BNC1, DHX32,* ENSCHIG00000003537, ENSCHIG00000015469, ENSCHIG00000016273, ENSCHIG00000017364, ENSCHIG00000026346
M2		*RRAS, MAP3K6, PLCD1, CAMK1D, NOTCH1, IL17RA, MSRB1, WFS1, MTHFD1L, PLPP2, DCXR, VSIR, CPT1A, RPS6KA2, USP31, PYGL*
M3		*GSE1, KCNA4, MAPKBP1, MBOAT7, MEAK7, NIPAL1, PISD, RAB40B, RIPOR3, SLC52A3, SLFN14, TIAM1, TTYH1, ZNF536, ATP13A4, BAHD1, CNPPD1*, ENSCHIG00000004635, ENSCHIG00000016460, ENSCHIG00000018407, ENSCHIG00000024862, ENSCHIG00000026843
M4	downregulated at 90 dpp	*LIMD1, DAB2, FGFR1, ARHGAP22, MXRA8, AGO4*, ENSCHIG00000020774, ENSCHIG00000022804
M5		*HDAC9, MAPT*
M6	upregulated at 120 dpp	*GOLGA7B, ZMYM2, CCDC182, AGFG1*, ENSCHIG00000023128
M7	downregulated at 120 dpp	*TYK2, ARAP1, FXN, UGCG*, ENSCHIG00000011827
M8		*MED25, ADAMTSL2, DHDH, QSOX1, CYP27A1*
M9		*ALDOB, HOXA5, TRPV6, FZD10, PIGR, CDH1, VIPR2*

### The time series expression patterns of DEGs

**Fig. 5:**
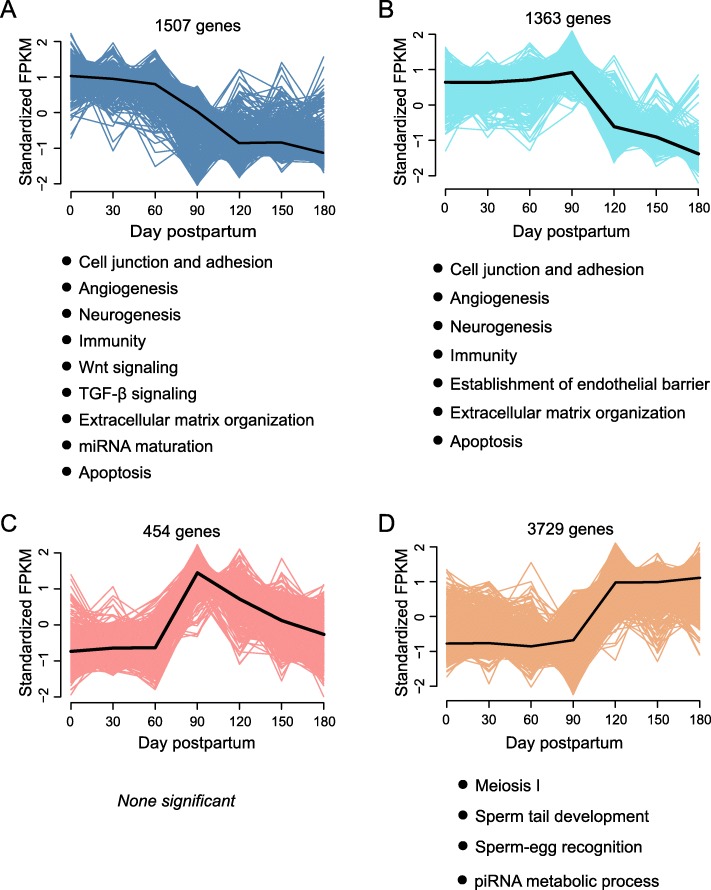
Fuzzy c-means clustering showing the temporal expression patterns of genes. Four patterns (**a**, **b**, **c** and **d**) were identified based on expression levels in seven developmental stages (0, 30, 60, 90, 120, 150 and 180 dpp). The cluster center for each pattern was highlighted as black lines in the plots. Gene number and the results of functional enrichment of each pattern was presented in the plots

We identified DEGs amongst adjacent age groups and traced their individual kinetic patterns at each age. These DEGs were clustered into four temporal patterns (Fig. 5, Additional file 6: Table S16), as determined by the fuzzy c-means clustering algorithm using the R package Mfuzz [5].

No significant change was observed in the gene transcriptional levels at 0–60 dpp in all patterns (Figs. 5a–d). The gene transcriptional levels in patterns A and B were repressed at 90–180 and 120–180 dpp, respectively (Fig. 5a and b). By contrast, patterns C and D exhibited activated gene expression at 90 (Fig. 5c) and 120 (Fig. 5d) dpp, respectively. Gene expression decreased at 120–180 dpp (pattern C, Fig. 5c) and was maintained at 150–180 dpp (pattern D, Fig. 5d).

Unsurprisingly, most DEGs were clustered into pattern D (3729 genes). The functional terms enriched in pattern D included *piRNA metabolic process, axonemal dynein complex assembly, spermatid nucleus differentiation, motile cilium assembly, cilium movement, synapsis, flagellated sperm motility, sperm–egg recognition* and *male meiotic nuclear division* (Fig. 5d, Additional file 7: Table S19), which were involved in spermatogenesis processes. The terms associated with cell junction, neurogenesis, muscle morphogenesis, immunity, regulation of Wnt signalling, regulation of TGF-β signalling, extracellular matrix organization, miRNA maturation, apoptosis and angiogenesis were enriched in pattern A (Fig. 5a, Additional file 7: Table S17).

A total of 1363 DEGs were clustered in pattern B (Fig. 5b), and their functional enrichment was similar to pattern A, whereas the terms associated with the growth of endothelial barrier were enriched in pattern B (Additional file 7: Table S18). The genes distributed into all the patterns and the functional enrichment results are listed (Additional file 6: Table S16 and Additional file 7: Tables S17-S19).

### Expression of testis development-related genes

We investigated the changes in the transcriptional level of well-documented genes, which were involved in the male reproductive process (Table 2). Deleted in azoospermia-like (*DAZL*) and deleted in azoospermia-associated protein 1 (*DAZAP1*) genes were upregulated at 90 dpp. Protamine 2 (*PRM2*), *PRM3* and transition protein 2 (*TNP2*) genes were upregulated at 120 dpp, and *TNP1* was upregulated at 90 and 120 dpp. The results of WGCNA showed that *PRM2* and *PRM3* were present in M6, which further suggested the active expression of protamines at 120 dpp. Synaptonemal complex protein 2 (*SYCP2*) was upregulated at 90 and 120 dpp, whereas *SYCP3* was upregulated only at 120 dpp.

**Table 2:** The expression of well documented genes which have important roles in male reproduction

Gene	D90 vs. D60	D120 vs. D90
*DAZAP1*	not significant	upregulated
*DAZL*	upregulated	not significant
*PRM2*	not significant	upregulated
*PRM3*	not significant	upregulated
*SYCP2*	upregulated	upregulated
*SYCP3*	not significant	upregulated
*TNP1*	upregulated	upregulated
*TNP2*	not significant	upregulated

We compared the results of previous studies with the results of the present study to explore the gene expression changes that were common in early-puberty animals and found some commonalities (Table 3). The results show that certain genes related to testicular development, such as *ODF2*, *STRA8*, *SOX9*, *MAPK6*, *STK36*, *PRKCQ*, *AMH*, *SOX9*, *VIM*, *SMO*, *PTCH1*, *PTCH2*, *GAS1*, *GLI1*, *WNT5A*, *WNT6*, *MAP3K1*, *MAPK12*, *MAPK14* and *JUN*, have similar expression trends in these studies.

**Table 3:** The expression of genes compared with other studies

Gene	Expression in reference study	Expression in present study	Reference
*ODF2*	upregulated from 2 months old to 4 months old and from 4 months old to 6 months old	upregulated from 60 dpp to 120 dpp and from 120 dpp to 180 dpp	[6]
*STRA8*	upregulated from 2 months old to 4 months old	upregulated from 60 dpp to 120 dpp	
*SOX9*	downregulated from 2 months old to 4 months old	upregulated from 0 dpp to 60 dpp but downregulated from 60 dpp to 120 dpp	
*MAPK6*	upregulated from infant (6 days postnatal) to juvenile (4 weeks old)	upregulated from 0 dpp to 120 dpp	[7]
*STK36*			
*PRKCQ*			
*AMH*	downregulated from infant (6 days postnatal) to juvenile (4 weeks old)	downregulated from 0 dpp to 120 dpp	[7]
*SOX9*			
*VIM*			
*SMO*			
*PTCH1*			
*PTCH2*			
*GAS1*			
*GLI1*			
*WNT5A*			
*WNT6*			
*MAP3K1*			
*MAPK12*			
*MAPK14*			
*JUN*			

### Quantitative real-time PCR (qRT-PCR)

**Fig. 6:**
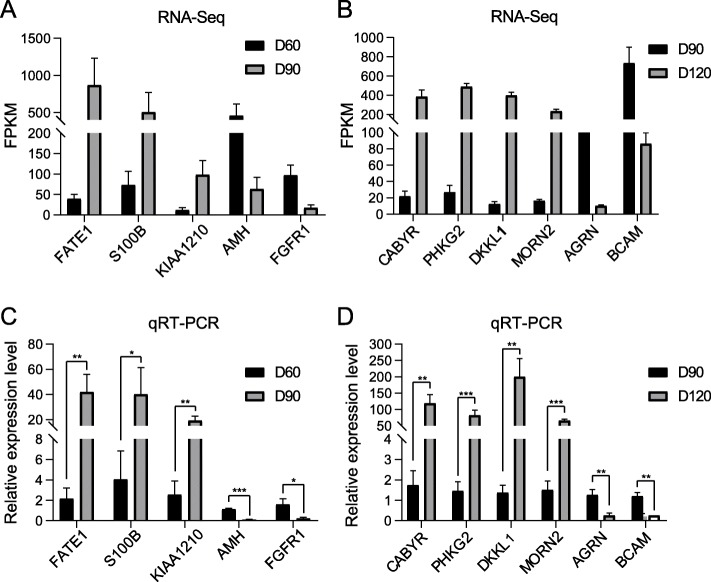
Validation of microarray results by qPCR analysis. **a** and **c** showed the results of RNA-seq and qRT-PCR of DEGs of D90 vs. D60. **b** and **d** showed the results of RNA-seq and qRT-PCR of DEGs of D120 vs. D90. The goat *ACTB* gene was used as reference gene. Data are shown as *means* ± *SD* (*n* = 3). *: *p* < 0.05. **: *p* < 0.01. ***: *p* < 0.001

To verify the accuracy of the transcriptome sequencing, 11 DEGs were randomly selected and used for qRT-PCR test. The results showed that the expression of these 11 DEGs in qRT-PCR test was consistent with the results of transcriptome sequencing (Fig. 6), which proved the reliability of the high-throughput RNA-Seq data.

## Discussion

### Yiling goats have a very early puberty

In this study, the spermatozoa were observed in the testes of 90-day-old Yiling goats (Fig. 1e), and the transcription levels of spermatogenesis-related genes also increased (Fig. 3b). Results showed that the age at puberty of Yiling goats was about 90 days old. Studies show that goats of different breeds have great diversity in age at puberty. For example, the puberty of Nubian goats, Boer goats and Saanen dairy goats are at 210, 157 and 147 days old, respectively [8, 9]. Evidently, the age at puberty of Yiling goat is shorter than other goat breeds. Thus, Yiling goats can be used as an animal model for early puberty research.

### Developmental stages of testis

Based on the PCA and correlation analysis of gene expression, early-puberty Yiling goats underwent three stages of testicular development (0–60, 60–120 and 120–180 dpp). The testis weight and spermatogenesis analysis results also reinforced this result. During the first stage (0–60 dpp), the testicular weight slowly increased, and no sperm cell was detected. In the second stage (60–120 dpp), the testicular weight and the seminiferous tubule diameter rapidly increased, and the seminiferous epithelium markedly thickened. In the third stage (120–180 dpp), increased testicular weight and seminiferous tubule diameter, slightly thickened seminiferous epithelium and a complete sequence of spermatogenesis were observed (Fig. 1b, e and f).

Two major changes in testicular development were also observed in sheep and salmon. In a study on Ghezel sheep (an early-puberty native Iranian sheep), the number of spermatogonia in seminiferous tubules increases remarkably from the age of 3 months, and spermatocyte and seminiferous tubule lumen are apparently observed from the age of 4 months [10]. Some low vertebrates have similar phenomena. The development of salmon testis after birth has three stages (i.e. developmental quiescent, prepuberty and puberty stages), and the regulation network of microRNAs and mRNAs has distinct characteristics in these three developmental stages [11]. Studying the changes in gene expression during the transition of developmental stages is important to understand the testicular development of early-puberty animals.

### Dynamic changes in genes and pathways during testicular development in early-puberty goats

#### Testicular growth before 60 dpp

In this study, the genes involved in organ growth were highly expressed from 0 dpp to 60 dpp. The expression of these genes is beneficial to the growth of blood vessel, nerve, epithelium, muscle and other tissues; organ size control; cell growth regulation; cell junction and adhesion. The growth of seminiferous tubules before puberty is reflected in their increase in length [12]. We found that testicular weight did not increase substantially from 0 dpp to 60 dpp (Fig. 1b), probably because the increase in seminiferous tubule length did not contribute significantly to the increase in testicular weight.

#### Dynamic changes in genes and pathways after the onset of puberty at 90 dpp

At 90 dpp, the occurrence of the first transition of testicular development, the downregulation of organ growth-related genes and the upregulation spermatogenesis-related genes were observed (Figs. 3b and, Fig. 5a, Additional file 3: Figure S1).

The blood–testis barrier is the key to protect spermatogenic cells and maintain the immune-privileged environment of seminiferous tubules. The cell junction between Sertoli cells is the main component of the blood–testis barrier [13]. In this study, we found that the genes involved in cell junction formation (e.g. *ITGA2, ITGA4* and *ITGA6*) were downregulated at 90 dpp, and this trend was consistent with the function of these genes.

The genes in the TGF-β signalling pathway are involved in cell growth and differentiation in mammals. Amongst these genes, *AMH* promotes testicular development by inducing Mullerian tube degeneration in the embryonic stage and maintains high expression level before puberty to maintain testicular growth and development [14]. In the current study, we found that *AMH* and some other genes involved in the TGF-β signalling pathway were downregulated at 90 dpp, and their expression patterns accorded with their important role in promoting testicular growth.

The testis is innervated by cholinergic and peptidergic nerves. The nerve terminals are distributed in the tunica albuginea, interstitial connective tissue, blood vessels and seminiferous duct wall. These nerve terminals form synaptic connections with Sertoli and Leydig cells. The nerves distributed in the testis regulate spermatogenesis [15, 16]. In this study, we found that neurogenesis-related genes were highly expressed in the testicular growth stage, and their transcriptional levels decreased in the transition and the spermatogenesis stages. These results corroborated that the normal spermatogenesis in testis needed the support of the nervous system. Before puberty, the middle testicular nerve developed rapidly and laid a foundation for the vigorous development of spermatogenesis during puberty.

The interstitium around the testicular seminiferous tubules in adult mammals has a large number of capillaries, most of which are formed before puberty [17]. The periphery of seminiferous tubules is covered by tubular myoid cells, which participate in the transport of sperm and testicular fluid in seminiferous tubules by contraction. These cells can also secrete many substances, including fibronectin, collagens I and IV, proteoglycan, other extracellular matrix components and growth factors, such as TGF-beta and IGF-1 [18]. In addition, myoid cells contain androgen receptors and participate in retinol processing [18]. The vascular development- and muscle growth-related genes were upregulated during the transitional and spermatogenesis stages. This finding indicated that vascular and muscle tissues provided the integrity of spermatogenic tubules and participated in testicular growth and spermatogenesis.

#### Downregulation of immune-related genes after 120 dpp

After 120 dpp, the transcriptional level of organ growth-related genes decreased gradually, whereas the expression of spermatogenesis-related genes began to flourish (Additional file 2: Tables S14 and S15). The upregulated spermiogenesis-related genes during this period were involved in meiosis, sperm tail development and sperm maturation. The haploid spermatocyte and the sperm cell surface proteins in animal organisms have strong immunogenicity. The testis is an immune-privileged tissue, but its internal environment has a weak immune response to autoantigens. Thus, the testis can avoid autoimmune diseases [19]. The results of this study supported the theory that the transcription level of immune-related genes decreases sharply with vigorous spermatogenesis from 120 dpp to 180 dpp (Additional file 2: Tables S14 and S15). The nuclear factor kappa-B (NF-kappa-B), a key mediator of inflammatory response, regulates inflammatory response by inducing the expression of various proinflammatory factors (including various cytokines and chemokines) and plays a key role in the activation and differentiation of innate immune cells and inflammatory T cells, which regulate many aspects of innate and adaptive immune functions [20]. Herein, we found that the transcription level of genes involved in the NF-kappa B signalling pathway dropped sharply at 120 dpp. We speculated that the NF-kappa B signalling pathway may play an important role in the testicular immune system before puberty. The body maintained the immune-privileged characteristics of the testis during the period of spermatogenesis by reducing the expression of the NF-kappa B signalling pathway.

In this study, results showed that the postnatal development of goat testis went through three stages. The first stage was the testicular growth stage. In this stage, the genes involved in the growth of nerve, blood vessel and cell junction were highly expressed, which prepared for the growth of seminiferous tubules, the development of spermatogenic epithelium and spermatogenesis. The next stage was the transitional stage. At this stage, a gradual decrease in the growth-related gene transcription levels, a gradual elevation in the spermatogenesis-related gene transcription levels and a gradual shift in the focus of testicular development into spermatogenesis were observed. In the spermatogenesis stage, spermatogenesis-related genes were highly expressed, and the testicular growth and immune-related genes were downregulated, thereby creating an immune-privileged environment for spermatogenesis.

### Key genes involved in testicular development transition

In this study, we used the WGCNA to construct co-expression networks with DEGs (M1–M3 for the upregulated genes from 60 dpp to 90 dpp, M4–M5 for the downregulated genes from 60 dpp to 90 dpp, M6 for the upregulated genes from 90 dpp to 120 dpp and M7–M9 for the downregulated genes from 90 dpp to 120 dpp), and the hub genes in each network were predicted. Most of the 84 hub genes were known spermatogenesis-, cell signal transduction- and immunity-related genes or genes with other known functions (Table 1). However, the function of some hub genes in testicular development is not clear.

The mutations in *DHX37*, the homologous gene of *DHX32*, are associated with XY gonadal dysgenesis [21]. Therefore, we speculated that *DHX32* may play a physiological role in gonadal development and spermatogenesis, but further research is needed. The mutations in *ART3* cause nonobstructive azoospermia [22], but the specific mechanism is not clear. *TDRKH* encodes a mitochondrial protein, which is involved in piRNA biogenesis and mediates transposon inhibition in meiosis [23]. Therefore, the role of *TDRKH* and piRNA in spermatogenesis deserves further study. The *MEAK7* gene encodes a mammalian target of rapamycin (mTOR)-associated protein, which activates the mTOR signalling pathway to regulate cell proliferation and migration in mammalian cells [24]. These genes were upregulated at 90 dpp (presented in M1–M3) in the present study. Therefore, their functions in spermatogenesis and other life activities needed further study.

As a member of the GTPase-activating protein family, the Rho GTPase-activating protein 22 (*ARHGAP22*) mediates the capillary formation of endothelial cells, thereby playing an important role in angiogenesis [25]. The matrix-remodelling-associated protein 8 (*MXRA8*) is known as a receptor of viral infection, but its role in cell adhesion and angiogenesis has been gradually discovered in recent years [26]. The microtubule-associated protein tau (MAPT) is involved in the establishment and maintenance of neuronal polarity and mutations in its gene led to nervous system diseases, such as Alzheimer’s disease, Pick’s disease, frontotemporal dementia and corticobasal degeneration [27–29]. The Golgin A7 family member B (*GOLGA7B*) has a role in cell adhesion, including desmosome assembly and other processes [30]. The downregulation of *ARHGAP22*, *MXRA8* and *MAPT* at 90 dpp (presented in M4–M5) and the upregulation of *GOLGA7B* at 120 dpp (presented in M6) may reflect the complex regulation of angiogenesis-, cell junction- and neurogenesis-related processes in the testis during puberty.

Some lysine residues in the N-terminal region of the core histone undergo reversible acetylation during spermatogenesis to promote histone–protamine replacement and allow the chromosome to shrink to a greater degree [31]. However, histone deacetylase blocks histone acetylation and inhibits histone–protamine replacement [32]. The histone deacetylase 9 (*HDAC9*) gene was downregulated at 90 dpp (presented in M5; Table 1), and its downregulation was conducive to the vigorous development of spermatogenesis in this period. In mammals, the argonaute RISC component 4 (AGO4) is abundantly expressed in the nucleus of spermatocyte during the prophase I of meiosis, and the *Ago4*^*−/−*^ spermatogonia initiate meiosis earlier than the wild-type *Ago4*^*+/+*^ probably because of small RNA pathways [33]. In the present study, the *AGO4* gene was significantly downregulated at 90 dpp (presented in M4). This occurrence may be one of the reasons why early-puberty goats initiate spermatogenesis earlier than late-maturing goats.

The zinc finger MYM-type containing protein 2 (*ZMYM2*) was significantly upregulated in the testes at 120 dpp (Table 1). Although the function of this gene has not been reported, another member of the gene family, *ZMYM3*, has been involved in the regulation of spermatogenesis and cell cycle [34]. Similar to *ZMYM2*, the function of coiled-coil domain containing protein 182 (*CCDC182*) gene is unclear, but the CCDC42 protein is expressed in the manchette, connecting piece and tail of spermatids [35]. The mutations in *CCDC42* cause sperm decapitation and impaired sperm motility [36]. The AGFG1 (previously known as HRB) protein may localise to the cytosolic surface of proacrosomic transport vesicles and is required for the fusion of proacrosomic transport vesicles into single large acrosome vesicles [37, 38]. These genes were upregulated at 120 dpp (presented in M6; Table 1). Considering the potential relationship between their known functions and life activities (such as spermatogenesis, androgen synthesis and secretion), these genes may be novel genes that play important roles in the testis at the puberty stage.

We also investigated the transcriptional changes in some well-documented genes (Table 2). The coding product of *DAZL* is a germ cell-specific RNA-binding protein that may play a role in sex differentiation and spermatogenesis at the level of translation initiation [39, 40]. In this process, the DAZAP1 protein may interact with the coding product of *DAZL* [39]. *PRM2*, *PRM3*, *TNP1* and *TNP2* mediate the replacement of histone in the sperm nucleus [41–43]. Additionally, synaptonemal complex proteins are important components of the synaptonemal complex, and the null mutations of their genes usually cause azoospermia with meiotic arrest [44, 45]. In the present study, *SYCP2* was upregulated at 90 and 120 dpp, whereas *SYCP3* was only upregulated at 120 dpp (Table 2). *PRM2, PRM3* and *TNP2* were upregulated at 120 dpp, whereas *TNP1* was upregulated at 90 and 120 dpp (Table 2). Moreover, *DAZL* and *DAZAP1* were upregulated at 90 dpp (Table 2), which corresponded to the onset of spermatogenesis in testis.

### Comparison with other studies

In early-puberty goat with an age at puberty close to the Yiling goat (about 4 months old), several upregulated spermatogenesis-related genes and downregulated organ growth-related genes are observed at ages of 2–4 months [6]. For example, *ODF2*, whose coding product constitutes the sperm tail axon [46], is upregulated at 2–4 and 4–6 months old. Stra8 is a vertebrate-specific cytoplasmic factor that acts as a meiosis-inducer required for the transition into meiosis in response to retinoic acid [47], and *STRA8* is significantly upregulated at 2–4 months old. *SOX9,* which is the gene for sex determination and the early testicular growth factor, is downregulated at 2–4 months old. Interestingly, identical results were observed in our study (Table 3). In the present study, *ODF2* was significantly upregulated from 60 dpp to 120 dpp and from 120 dpp to 180 dpp, *STRA8* was upregulated from 60 dpp to 120 dpp, and *SOX9* was upregulated from 0 dpp to 60 dpp but downregulated from 60 dpp to 120 dpp. The common expression pattern of genes *ODF2, STRA8* and *SOX9* may partially explain the early puberty of these goats.

We also compared the results of this study with similar studies in other species, such as mice. In a previous study, certain testicular growth biomarkers (including genes *AMH, SOX9, WT1* and *VIM*) are downregulated at 4 weeks old compared with those at 6 days postnatal [7]. Moreover, some genes in MAPK, Hedgehog and Wnt signalling pathways present different regulation patterns. For instance, *SMO, PTCH1, PTCH2, GAS1, GLI1, WNT5A, WNT6, PRKCI, MAP3K1, MAPK12, MAPK14* and *JU* are downregulated, whereas *MAPK6, STK36* and *PRKCQ* are upregulated at 4 weeks old [7]. This result was consistent with the results of the present study (Table 3). Considering that mice are early-puberty and fertile mammals, these results can reflect some of the common characteristics of early-puberty animals to some extent.

By comparing the above studies, we have found that the expression patterns of several genes are similar during the testicular development of early-puberty goats and mice. However, we cannot directly determine whether the expression pattern of these genes is unique to early-puberty goats because of the lack of relevant studies on late-maturing goats. Therefore, more research on testicular development of late-maturing goats is needed to ascertain this assumption.

## Conclusions

Our results showed that the Yiling goats entered the puberty stage at a very early age (90 dpp), as evidenced by the dramatic increase in testicular weight and the presence of sperm cells in the seminiferous tubules. Generally, early-puberty Yiling goats from birth to 180 dpp experienced three stages of testicular development, namely, the growth, transition and spermatogenesis stages. During this period, spermatogenesis-related genes were upregulated, whereas neurogenesis-, angiogenesis-, cell junction-, muscle- and immune-related genes were downregulated. Additionally, we found several novel hub genes, including *DHX32, ART3, TDRKH, MEAK7, ARHGAP22, MXRA8, MAPT, GOLGA7B, HDAC9, AGO4, ZMYM2, CCDC182* and *AGFG1,* which may play key roles in spermatogenesis, androgen synthesis and secretion, angiogenesis, cell junction and neurogenesis for the puberty development of testis. Moreover, we compared the results of the present study with previous studies on goat or other mammals, and the regulation patterns of some genes shared in these early-puberty mammals (including *ODF2, STRA8, SOX9, AMH, SOX9, WT1, VIM*, *SMO, PTCH1, PTCH2, GAS1, GLI1, WNT5A, WNT6, PRKCI, MAP3K1, MAPK12, MAPK14, JUN, MAPK6, STK36* and *PRKCQ*) were observed. Overall, the multipathway synergy promotes testicular transition from growth to spermatogenesis in early-puberty goats and may be a common rule shared by mammals. This in-depth analysis of the transition from testicular growth to spermatogenesis provides important clues for understanding the mechanisms underlying early puberty.

## Methods

### Sampling

For this study, 21 male Yiling goats with average ages of 0, 30, 60, 90, 120, 150 and 180 days old were randomly selected and purchased from the Changyang Yongxing Ecological Husbandry Co., Ltd. (Yichang, Hubei, China). Each age group comprised three goats (Additional file 8: Table S20). The goats were raised in the same environmental conditions. The goats were sedated intramuscularly by using 0.1 ml/kg su mian xin also known as xylazine hydrochloride (Shengda, Changchun, Jilin, China), and the testicles were surgically collected and weighed using an electronic balance. One testis was fixed with 4% paraformaldehyde, and the other was frozen immediately in liquid nitrogen and then stored at − 80 °C.

### Growth model of body and testis

The nonlinear growth model used to describe the growth curves of Yiling goat is shown in Fig. 1. The logistic function was used to model the relationship of age with body and testicular weights by using the SAS program (SAS Institute, 2002), and the parameters were estimated.

### HE staining of testicular tissue

The testis was washed with 0.9% saline, fixed with 4% paraformaldehyde for 48 h at room temperature and embedded for further histologic analysis. The tissues were sliced into sections of 5 μm thick and stained with HE, and the morphology of the testis was observed under the Eclipse-Ci™ microscope (NIKON, Chiyoda, Tokyo, Japan).

### Library construction and RNA sequencing

Total RNA was extracted from the testicular tissue, and ribosomal RNA was removed using the Ribo-Zero™ kit (Epicentre, Madison, WI, USA). The RNA was interrupt and cDNA was then synthesised by reverse transcription. After polymerase chain reaction amplification and purification by using the Qubit® dsDNA high-sensitivity assay kit, the fragment length of approximately 200 bp was chosen for library construction by using the NEBNext® Ultra™ RNA library prep kit. The libraries were paired-end sequenced (PE150, the length of reads was approximately 150 bp) using the Illumina HiSeq X Ten platform at the Megagenomics Company (Beijing, China). The raw data have been deposited in the Genome Sequence Archive [48] under accession number CRA002191. The quality of the raw data was determined using the FastQC software [49], and the adapters were trimmed using the Trimmomatic [50].

### Mapping and assembly

Clean high-quality paired-end reads were mapped to a goat reference genome (GCA_001704415.1, ftp://ftp.ensembl.org/pub/release-94/fasta/capra_hircus/dna/Capra_hircus.ARS1.dna.toplevel.fa.gz) by using the Hisat2 software (V2.1.0) [51] with default parameters. The annotation for protein-coding genes were downloaded from the Ensembl database (ftp://ftp.ensembl.org/pub/release-94/gtf/capra_hircus/Capra_hircus.ARS1.94.gtf.gz). The transcripts were assembled using the StringTie program (V1.3.4) [51] with parameters for mRNA analysis, including ‘-e’ and ‘-G’.

### PCA and correlation analysis of gene expression level

The abundance of each gene was defined using the fragments per kilobase of exon per million fragments mapped (FPKM), and the FPKM matrix of all the genes was inputted for the analyses of this section. PCA and correlation analysis were performed using the R language [52], and results were visualised using the R packages, namely, ggplot2 [53] and pheatmap [54].

### Differential expression analysis

The read count matrix of genes was generated using the HTSeq software (version 0.11.1) [55]. DEGs were calculated using the DESeq2 package [56]. We treated the samples at the same age as replicates and compared the adjacent ages (i.e. D30 vs. D0, D60 vs. D30, D90 vs. D60, D120 vs. D90, D150 vs. D120 and D180 vs. D150) to obtain the DEGs. The *P* value for each gene was obtained based on the model of negative binomial distribution. The fold changes were also estimated using the DESeq2 package. The *P* values were adjusted using the Benjamini and Hochberg method [57]. The screening criteria for DEGs were *q* < 0.05 and |fold change| ≥ 2. We then combined the DEGs in different groups to one DEG union set and performed the subsequent analysis.

### WGCNA

Gene co-expression networks were constructed using the R package WGCNA (v1.67) [58]. The FPKM values of the upregulated and downregulated genes of D90 vs. D60 and D120 vs. D90 were inputted for the analysis. The modules were clustered using the automatic network construction function, blockwiseModules, with default settings. An eigengene was chosen for each gene module to represent the expression pattern. Cytoscape software [59] was used to visualise the gene co-expression networks. Degree, as one important topological property and defined as the number of links to gene nodes, was used to define the hub genes of the networks.

### Time series expression patterns of genes

The Mfuzz (V2.44.0) [5], an R package for the noise-robust soft clustering of gene expression time series data, was used to analyse the expression patterns of DEGs by following the fuzzy c-means clustering algorithm. The FPKM matrix of the DEG union set described in the section ‘Differential expression analysis’ was inputted for this analysis. We measured the minimum intercluster Euclidian centre distance when the number of clusters 50 ≥ c ≥ 1 with up to 1000 iterations and repeated the analysis 10 times to estimate the number of cluster centres. The greatest drop in the minimum distance was observed when *c* = 4.

### GO enrichment analysis

The PANTHER website (http://geneontology.org/) [60–62] was used to perform the GO enrichment analysis. The genes in each module of WGCNA (M1–M9) and in each pattern in the time series expression pattern analysis (patterns A–D) were used for GO enrichment analysis. The cow orthologues of the genes were used as input. The default background gene list was used in this analysis. The screening criterion for significantly enriched terms was false discovery rate (*FDR*) < 0.05.

### qRT-PCR test

The total RNA samples were reverse-transcribed using the PrimeScript RT reagent Kit with gDNA Eraser (Toyobo, Osaka, Japan). The reaction system contained 1 μg of RNA, 2 μl of 5 × RT Buffer, 0.5 μl of Primer Mix, 0.5 μl of Enzyme Mix and deionized water in a final volume of 10 μl. The reaction was carried out at 37 °C for 15 min and 98 °C for 5 min. The cDNA was diluted 10 times and then used as the template of qRT-PCR. The qRT-PCR reaction system contained 10 μl of 2 × Hieff® qPCR SYBR Green Master Mix (Yeasen Biotech, Shanghai, China), 2.5 μl of cDNA, 0.5 μl of upstream primer, 0.5 μl of downstream primer and deionized water in a final volume of 20 μl and the experiment was conducted using a DNA Engine Opticon™ 2 Real-Time System (Bio-Rad, USA) Meanwhile, the PCR was conducted at 95 °C for 5 min, followed by 40 cycles of 95 °C for 10 s, 60 °C for 20 s and 72 °C for 20 s. The gene expression level was determined by the 2^-ΔΔCt^ algorithm, and the goat beta-actin (*ACTB*) gene was used as an internal control [63]. Each sample had three biological replicates, and the gene expression level was presented as the means ± standard errors (*SE*) (*n* = 3). The primer sequences of the selected genes are shown in Table 4.

**Table 4:** Primer sequences for qRT-PCR test

Gene	Forward primer	Reverse primer
*ACTB*^a^	GTCACCAACTGGGACGACAT	CATCTTCTCACGGTTGGCCT
*FATE1*	GCATGAGGTCCCAGTACGAG	GTAGCGCAGACGCTCAGTAA
*KIAA1210*	TATGAAGGCACTGCTTGCCA	ACAGAACCCTCCAAACCTGC
*S100B*	ATATTCCGGGAGGGAAGGTG	GGTAACCATGGCAACGAAAGC
*AMH*	CATACCAGGCCAACAACTGC	CTGATGAGGAGCTTGCCTGT
*FGFR1*	AAATGCCCTTCCAGTGGGAC	AGGTGGCATAACGGACCTTG
*DKKL1*	AGGTGGTGGCATCCATTGAG	ATGATCCAGAAGGCCACTCG
*PHKG2*	AAGACGTCATTGGCAGAGGAG	GCGACCTGGCGAAGGATATG
*CABYR*	GCAGGACAACCACCACCATA	GGTCTTCTTGCTGTCGTCCA
*MORN2*	TGGTTTCGGAAGACTTGAGC	ACTTTGCCCCAGTTGGGAAT
*BCAM*	GACTACGTGTGCGTGGTGAA	GCTGCAAGTGGCTATCTCCT
*AGRN*	GTGAAGAATGGGGAAGCCGA	GTTCGCACACACTGCCATAC

^a^Gene *ACTB* was used as the reference gene

## Supplementary information

**Additional file 1: Table S1 (Sheet 1):** Information of genes clustered in M1. **Table S2** (**Sheet 2):** Information of genes clustered in M2. **Table S3** (**Sheet 3):** Information of genes clustered in M3. **Table S4** (**Sheet 4):** Information of genes clustered in M4. **Table S5** (**Sheet 5):** Information of genes clustered in M5. **Table S6** (**Sheet 6):** Information of genes clustered in M6. **Table S7** (**Sheet 7):** Information of genes clustered in M7. **Table S8** (**Sheet 8):** Information of genes clustered in M8. **Table S9** (**Sheet 9):** Information of genes clustered in M9. The tables showed the Ensembl ID, name, location and description of the genes.

**Additional file 2: Table S10 (Sheet 1):** GO enrichment result of genes clustered in M1. **Table S11 (Sheet 2):** GO enrichment result of genes clustered in M2**. Table S12 (Sheet 3):** GO enrichment result of genes clustered in M4. **Table S13 (Sheet 4):** GO enrichment result of genes clustered in M6**. Table S14 (Sheet 5):** GO enrichment result of genes clustered in M7**. Table S15 (Sheet 6):** GO enrichment result of genes clustered in M8.

**Additional file 3: Figure S1.** Gene modules identified by WGCNA and functional enrichment of downregulated genes of D90 vs. D60. (A) Hierarchical cluster dendrogram of downregulated genes of D90 vs. D60 obtained by clustering the dissimilarity based on consensus topological overlap. Modules corresponding to branches were labeled with colors indicated by the color bands underneath the tree. A total of two modules were identified. (B) Top twenty of functional enrichment results for M4. The top twenty GO terms with the lowest *FDR* were shown in the figure.

**Additional file 4: Figure S2.** Gene modules identified by WGCNA and functional enrichment of upregulated genes of D120 vs. D90. (A) Hierarchical cluster dendrogram of upregulated genes of D120 vs. D90 obtained by clustering the dissimilarity based on consensus topological overlap. Modules corresponding to branches were labeled with colors indicated by the color bands underneath the tree. Only one module was identified. (B) Top twenty of functional enrichment results for each module. The top twenty GO terms with the lowest *FDR* value were shown in the figure.

**Additional file 5: Figure S3.** Gene modules identified by WGCNA and functional enrichment of downregulated genes of D120 vs. D90. (A) Hierarchical cluster dendrogram of downregulated genes of D120 vs. D90 obtained by clustering the dissimilarity based on consensus topological overlap. Modules corresponding to branches were labeled with colors indicated by the color bands underneath the tree. A total of three modules were identified. (B) Top ten of functional enrichment results for M7 and M8. The top ten GO terms with the lowest *FDR* value were shown in the figure.

**Additional file 6: Table S16.** The list of genes distributed into each of the four temporal patterns.

**Additional file 7: Table S17 (Sheet 1):** Functional enrichment result of genes in pattern A. **Table S18 (Sheet 2):** Functional enrichment result of genes in pattern B. **Table S19 (Sheet 3):** Functional enrichment result of genes in pattern D.

**Additional file 8: Table S20.** Information of experimental animals and transcriptome assembly details.

## Data Availability

The data used for this paper are available in the article and its additional files and the raw data have been deposited in the Genome Sequence Archive [48] in BIG Data Center [64], Beijing Institute of Genomics (BIG), Chinese Academy of Sciences, under accession number CRA002191 that is publicly accessible at https://bigd.big.ac.cn/gsa. The goat reference genome (GCA_001704415.1, ftp://ftp.ensembl.org/pub/release-94/fasta/capra_hircus/dna/Capra_hircus.ARS1.dna.toplevel.fa.gz) and the annotation for protein-coding genes (ftp://ftp.ensembl.org/pub/release-94/gtf/capra_hircus/Capra_hircus.ARS1.94.gtf.gz) were all obtained from the Ensembl database.

## Abbreviations

dpp: day postpartum
HE: Haematoxylin and eosin
PCA: Principal component analysis
DEG: Differentially expressed gene
WGCNA: Weighted gene co-expression network analysis
GO: Gene ontology
*DAZL*: Deleted in azoospermia-like
*DAZAP1*: Deleted in azoospermia-associated protein 1
*PRM2*: Protamine 2
*TNP2*: Transition Protein 2
*SYCP2*: Synaptonemal Complex Protein 2
NF-kappa-B: Nuclear factor kappa-B
*ARHGAP22*: Rho GTPase-activating protein 22
*MXRA8*: Matrix-remodelling-associated protein 8
*MAPT*: Microtubule-associated protein tau
*GOLGA7B*: Golgin A7 family member B
*HDAC9*: Histone deacetylase 9
*AGO4*: Argonaute RISC component 4
*ZMYM2*: Zinc finger MYM-type containing protein 2
*CCDC182*: Coiled-coil domain containing protein 182
FPKM: fragments per kilobase of exon per million fragments mapped
*FDR*: False discovery rate
*ACTB*: beta-actin
SE: Standard errors
