# Supplementary figures and images for: Multipathway synergy promotes testicular transition from growth to spermatogenesis in early-puberty goats
Source: BMC Genomics. 2020 May 25;21:372. doi: 10.1186/s12864-020-6767-x (PMC7249689; doi:10.1186/s12864-020-6767-x)

A

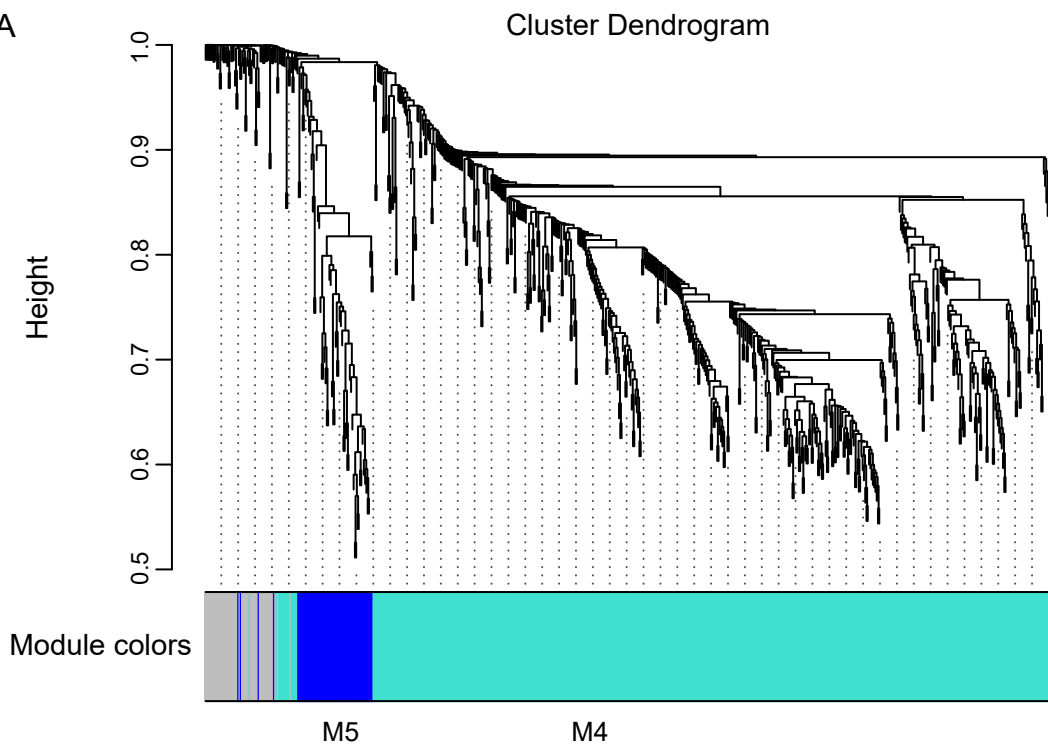

B

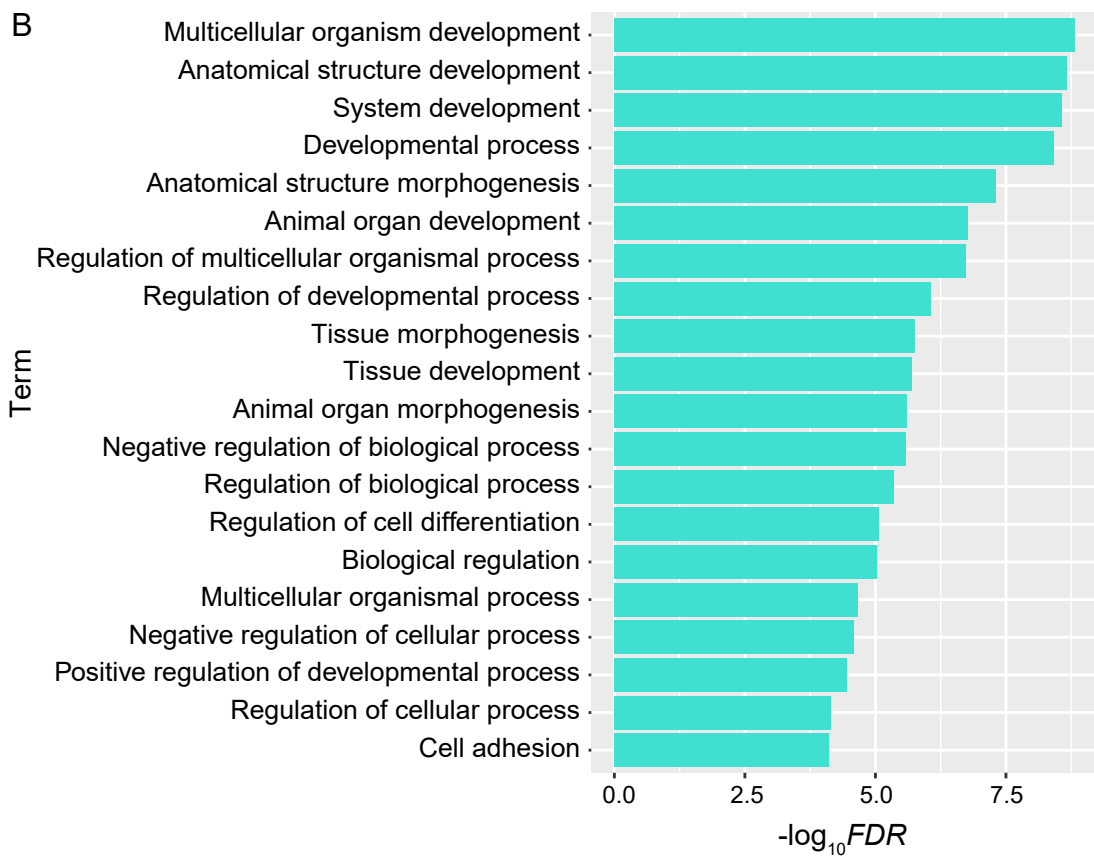

Supplement: Supplementary file 3 — Additional file 3: Figure S1. Gene modules identified by WGCNA and functional enrichment of downregulated genes of D90 vs. D60. (A) Hierarchical cluster dendrogram of downregulated genes of D90 vs. D60 obtained by clustering the dissimilarity based on consensus topological overlap. Modules corresponding to branches were labeled with colors indicated by the color bands underneath the tree. A total of two modules were identified. (B) Top twenty of functional enrichment results for M4. The top twenty GO terms with the lowest FDR were shown in the figure. [file 12864_2020_6767_MOESM3_ESM.pdf]

A

Cluster Dendrogram

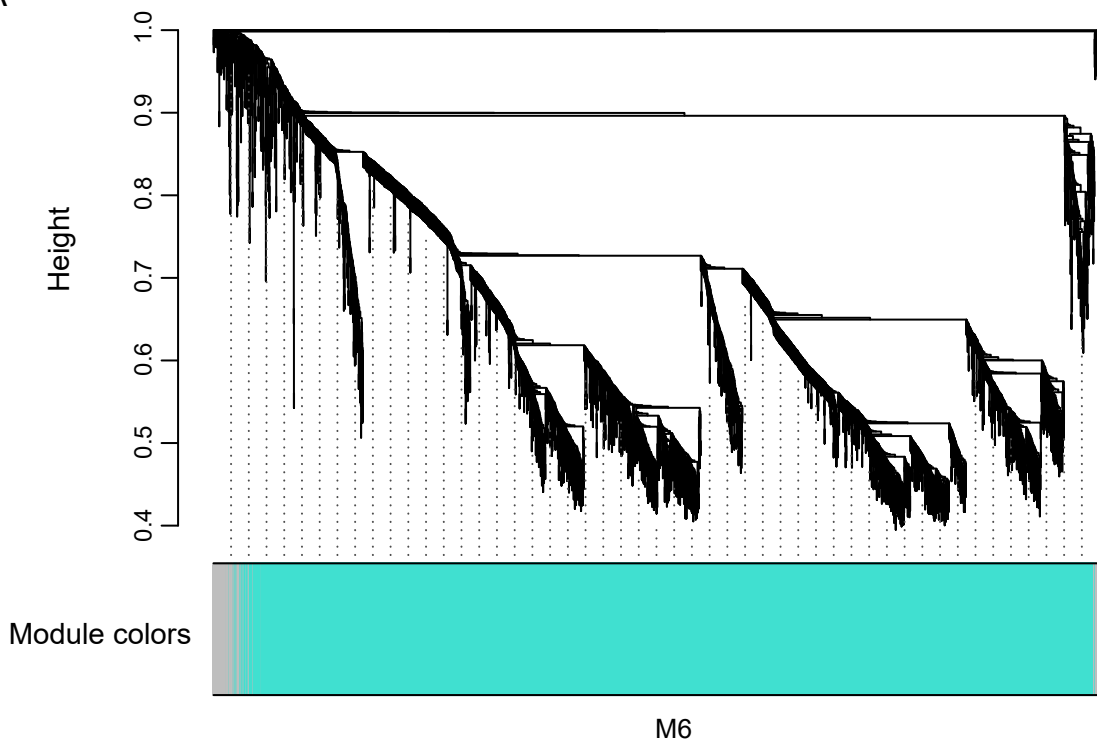

B

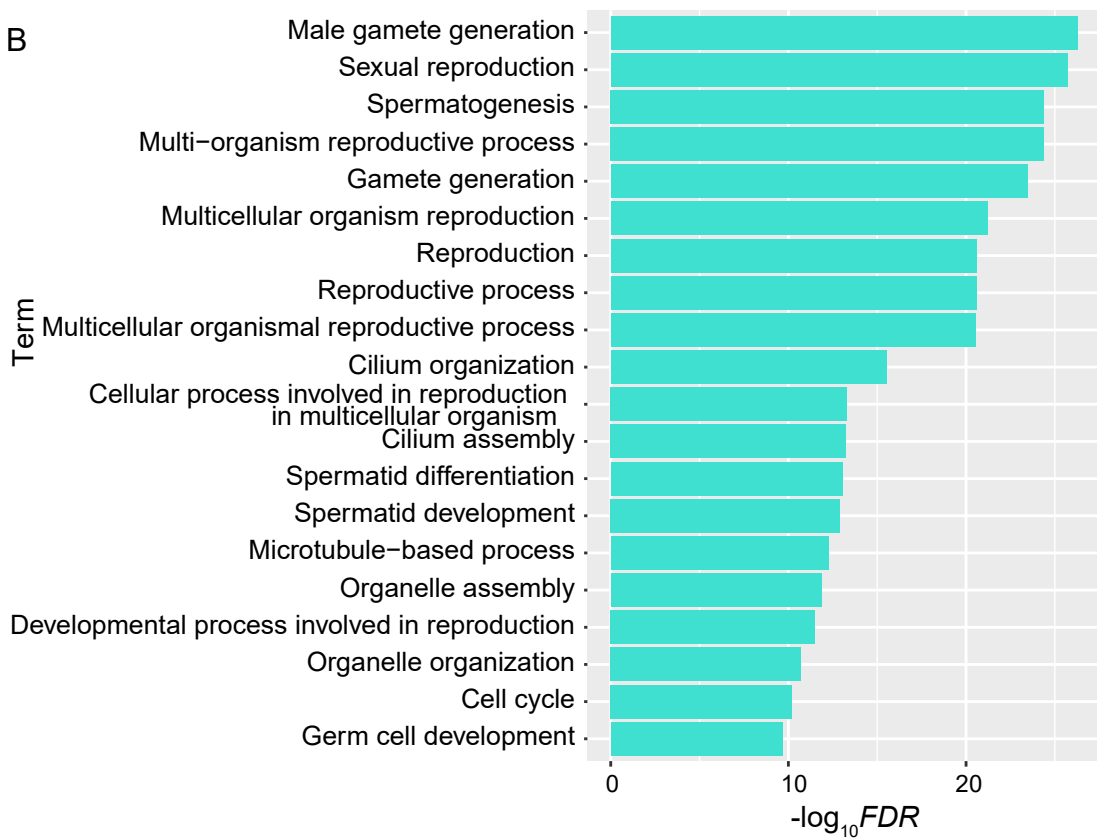

Supplement: Supplementary file 4 — Additional file 4: Figure S2. Gene modules identified by WGCNA and functional enrichment of upregulated genes of D120 vs. D90. (A) Hierarchical cluster dendrogram of upregulated genes of D120 vs. D90 obtained by clustering the dissimilarity based on consensus topological overlap. Modules corresponding to branches were labeled with colors indicated by the color bands underneath the tree. Only one module was identified. (B) Top twenty of functional enrichment results for each module. The top twenty GO terms with the lowest FDR value were shown in the figure. [file 12864_2020_6767_MOESM4_ESM.pdf]

A

Cluster Dendrogram

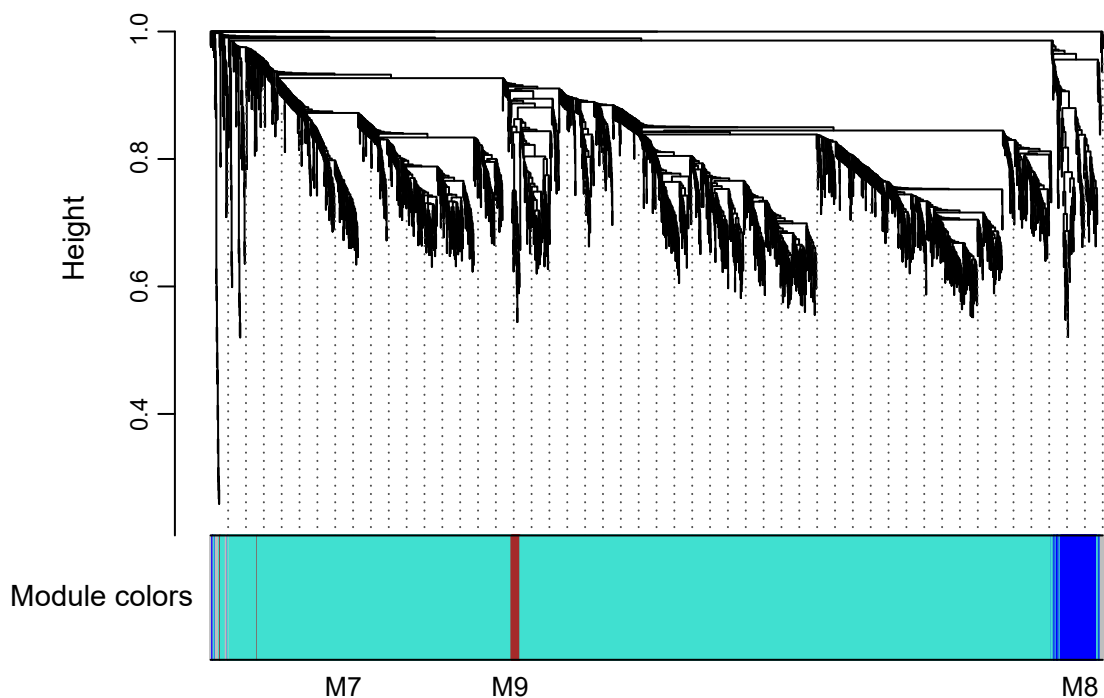

B

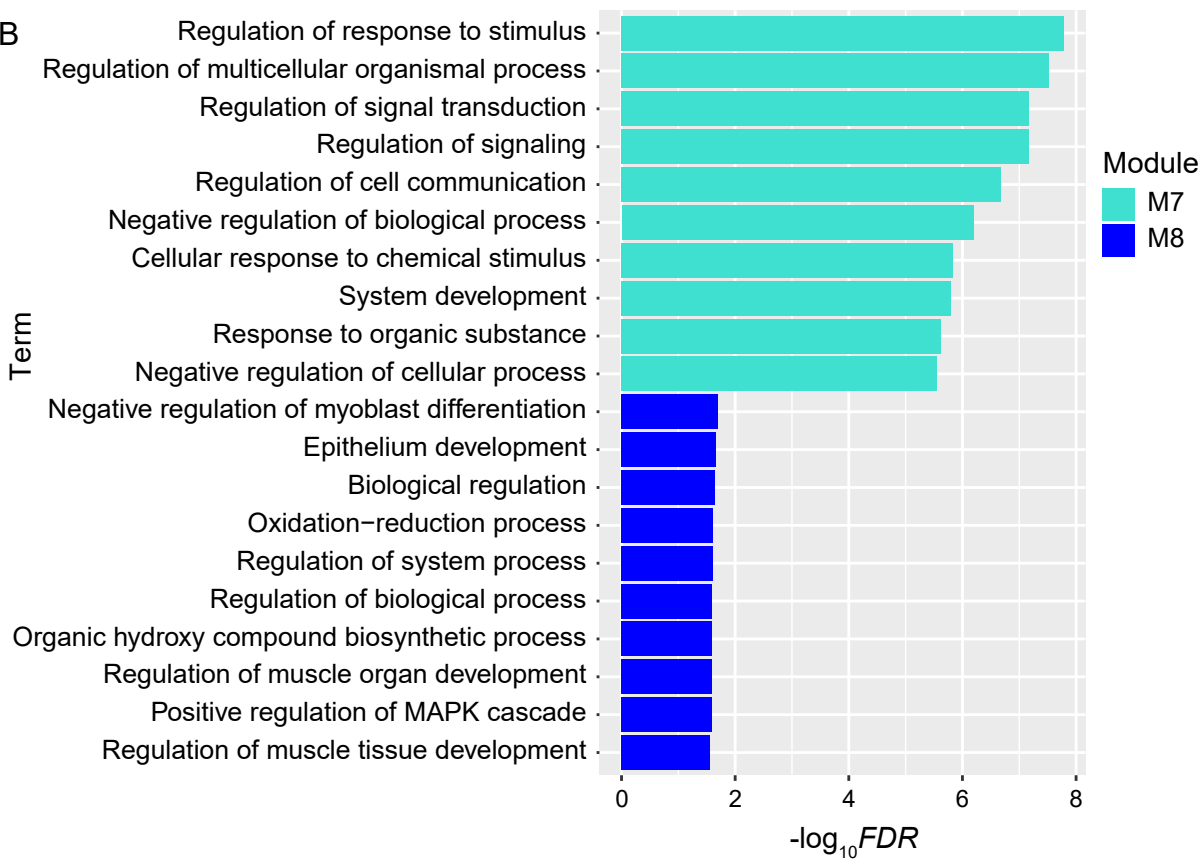

Supplement: Supplementary file 5 — Additional file 5: Figure S3. Gene modules identified by WGCNA and functional enrichment of downregulated genes of D120 vs. D90. (A) Hierarchical cluster dendrogram of downregulated genes of D120 vs. D90 obtained by clustering the dissimilarity based on consensus topological overlap. Modules corresponding to branches were labeled with colors indicated by the color bands underneath the tree. A total of three modules were identified. (B) Top ten of functional enrichment results for M7 and M8. The top ten GO terms with the lowest FDR value were shown in the figure. [file 12864_2020_6767_MOESM5_ESM.pdf]
